# Multi-Gas Analyzer Based on Tunable Filter Non-Dispersive Infrared Sensor: Application to the Monitoring of Eco-Friendly Gas Insulated Switchgears

**DOI:** 10.3390/s22228662

**Published:** 2022-11-09

**Authors:** Yera Kim, Sun-geun Goo, Jeong Sik Lim

**Affiliations:** 1 GHGs Metrology Team, Korea Research Institute of Standards and Science, Daejeon 34113, Korea; 2 Science of Measurement, University of Science & Technology, Daejeon 34113, Korea; 3 KEPCO Research Institute, Daejeon 58322, Korea

**Keywords:** multi-gas analyzer, tunable filter NDIR sensor, wide dynamic range, gas insulated switchgear, Novec-4710, SF_6_

## Abstract

This study presents a multi-gas analyzer based on tunable filter non-dispersive IR (TF-NDIR) sensors that operate with a wide dynamic range of wavelength and concentration. A pyroelectric sensor coupled with a microsized Fabry–Perot interferometer, namely a tunable filter, enables sensing within a narrowly selected wavelength band. Three detectors capable of tuning the bandpass wavelength with a range of 3.8–5.0 μm, 5.5–8.0 μm, and 8.0–10.5 μm are combined to encompass the entire mid-IR region. single-pass cell with an optical path length (OPL) of 5 cm and a multi-pass cell with an OPL of 10.5 m is selected to encompass a concentration range from ppmv to percent. The TF-NDIR sensors and gas cells can be reconfigured by manipulating the beam path. A homemade lock-in amplifier is used to enhance the signal-to-noise ratio 88 times greater than that of the bare signal. The performance of the gas analyzer is evaluated by measuring the SF_6_ and Novec-4710/CO_2_ mixture, which are the dielectric gas medium for a gas-insulated switch (GIS). The mixing ratio of the Novec-4710/CO_2_ mixture is measured within a range of 3–7% using premixes. The measurement precision is 0.72% for 0.5 s. Trace level measurements of Novec-4710, CO_2_, SF_6_, which are measurands for detecting gas leakage from the GIS, CO, and SO_2_ which are measurands for detecting product generated by the arc or thermal decomposition in the switching electrode, are conducted based on dynamic partial pressure adjustment using 1000 ppmv mother premixes in N_2_. The limit of detection is 54.7 ppmv for Novec-4710, 112.8 ppmv for CO, 118.1 ppmv for CO_2_, 69.5 ppmv for SO_2_, and 33.5 ppmv for SF_6_.

## 1. Introduction

Non-dispersive infrared (NDIR) spectroscopy is a commonly used method in the field of gas sensing [1,2,3]. A typical NDIR optical system comprises an infrared (IR) emitter, gas cell, optical band pass filter (OBPF), and detector. The broadband IR emission passes through the gas cell and the OBPF selects the gas species, which is measured using a broadly responding pyroelectric sensor. The NDIR sensor presents a quick response and sensitive detection to ensure an appropriate selection of the BPF. It is a non-contact gas detection system, exhibiting stability and robustness. Generally, the valid operation period of a NDIR sensor is longer than that of the contact combustion or electrochemical sensors [4,5]. Furthermore, its excellent detection sensitivity enables the detection of trace-level concentrations since most gases have a high line strength in the mid-IR (MIR) region, which is approximately 10~1000 times larger than that in the near-IR (NIR) regions [6,7,8]. The NDIR sensors can be implemented in various applications including heating, ventilation, and air conditioning (HVAC) [9], environmental diagnostics [10,11], medical diagnostics [12], breadth analysis [13,14], and trace gas sensing [15] owing to their robustness. The qualitative analysis of unknown mixtures faces certain limitations since the selectivity depends on the center wavelength and bandwidth of the OBPF. Typically, multiple measurands require an equal number of sensors [16,17,18]. Recently, major breakthroughs have been achieved in improving multi-gas detectability by combining multiple NDIR sensors [19] and in improving the sensitivity by using cutting-edge electrochemical sensors [20]. However, using multiple sensors increases the cost and does not present a complete dynamic range in wavelength. Therefore, this study presents a pyroelectric detector (PD) integrated with a microsized Fabry–Perot interferometer (FPI) to overcome this issue [21,22]. The center wavelength transmitted through the FPI filter was adjusted by varying the distance between the two reflectors, and the piezoelectric transducer (PZT) was electrically controlled. This aspect provided a dynamic selection of the center wavelength to avoid the use of multiple OBPFs, i.e., multiple NDIR sensors. An NDIR gas analyzer with a wide dynamic wavelength (3.8–10.5 μm) and concentration (ppmv to percent) range, that can adapt to various gas sensing applications, was employed in this study. The SF_6_ and Novec-4710/CO_2_ gas mixture, which are dielectric gas mediums for gas-insulated switchgears (GIS), were measured in various concentrations to comprehensively evaluate the performance of the analyzer. SO_2_ and CO are the byproducts generated by the SF_6_ and Novec-4710/CO_2_ mixture in the high-voltage arc of GIS and are considered chemical indicators for the health monitoring of GIS. They were also measured at the ppmv level. The mixing ratio of Novec-4710 and CO_2_ was approximately 5%, which is a typical mixing ratio for eco-friendly GIS, and was measured in a single-pass (SP) cell with an optical path length (OPL) of 5 cm. Other measurements were performed at trace levels for leak detection and health monitoring applications of the GIS at the ppmv level with a multi-pass (MP) cell with an OPL of 10.5 m.

## 2. Materials and Methods

### 2.1. Optical Layout

**Figure 1:**
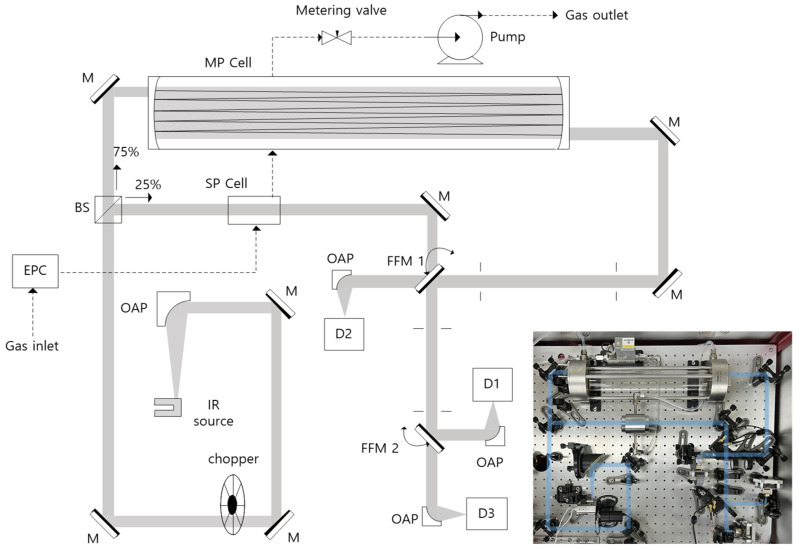
Optical layout and gas stream of the developed TF-NDIR spectrometer (EPC: electric pressure controller, M: mirror, FFM: flip flop mirror, OAP: off-axis parabolic mirror, BS: beam splitter, SP: single pass, MP: multi pass, D1: detector with wavelength tuning range (WTR) of 3.8–5.0 μm, D2: detector with WTR of 5.5–8.0 μm, D3: detector with WTR of 8.0–10.5 μm.

Figure 1 depicts the configuration of the optical layout of the proposed tunable filter NDIR (TF-NDIR) analyzer. Silicon carbide (SiC, Hawkeye Tech, IR-Si217, 37 W, T = 1385 °C, emissivity = 80%) was used as the thermal radiation source. The point light source, SiC, was irradiated in all directions. The SiC was placed at the focal point of the off-axis parabolic mirror (OAP, diameter = 50.8 mm, focal length = 101.6 mm) to collimate the light source. The collimated beam was modulated at a frequency of 20 Hz using an optical chopper (Thorlabs, (Newton, NJ, USA), MC1F2), and the power was split into a right angle with a split ratio of 25:75 for reflection and transmission. The reflected beam passes through the SP cell (OPL = 5 cm) bracketed by antireflection (AR) coated Zinc selenide (ZnSe). Furthermore, the transmitted beam is fed to a Herriot-type multi-pass (MP) cell (Thorlabs, HC10L-M02, OPL = 10.5 m, 28 reflections) with two gold-coated concave mirrors, which have a reflectance of over 95% within the wavelength range of 2–20 μm. The curvature of the reflectors partly compensates for the divergence of the collimated beam within the path inside the MP. The inlet and outlet windows of the MP were composed of AR-coated ZnSe, in which the transmission in 3–11 μm was over 90%. The optical transmittivity and reflectivity of the optical components of the MP presented a power throughput of ~50% at 3~11 μm. The beams that passed through the SP and MP were detected by three FPI-PD, which are denoted as D1 (Infratec, LFP-3850C-337, 3.8–5.0 μm), D2 (Infratec, FP-5580C-337, 5.5–8.0 μm), and D3 (Infratec, FP-80105C-337, 8.0–10.5 μm). The OAP (f = 25.4 mm) focused the beam in front of the detectors to enhance the detection sensitivity. The signal enhancement was 4–10 times greater than that of the bare cases. The gas cells and detectors were selected based on the vertical position of the gold mirror attached to the motorized flip-flop mount (FFM, Thorlabs, MFF101) located along the beam passes. Table 1 lists the combinations of the gas cell and detector, which were configured according to the FFM status. The “on” state corresponds to the FFM deviating from the optical path to cause the collimated light source to pass through unchanged. Conversely, the “off” state corresponds to the FFM coming up to the optical path to reflect the light source beam, thereby causing the optical path to change. The mirror surfaces were spatially aligned to bisect the volume of the light source beam intersection in order to prevent a lateral shift during reflection by the FFM. The gas cell can be selected based on the FFM1 status, passing through the SP cell when FFM1 is on and through the MP cell when FFM1 is off. The optical path length of the SP and MP cells differs by 210 times, and the appropriate cell is selected based on the sample concentration of the target measurement. FFM2 is used to determine the detector selection. When FFM2 is “on,” D3 is selected and when the FFM2 is “off”, D1 is selected. Lastly, D2 can detect only signals that passed through the MP cell with the FFM1 in the “on” state, regardless of the FFM2 status. An electronic pressure controller (EPC, Bronkhorst, EL-PRESS P-602CV, accuracy: ±1.75 Torr) was used to quantitatively fill the sample into the gas cells. The EPC monitors the downstream pressure for the PID control of the control valve EPC until the set point and monitoring pressure values match; the sample inside the cell is thus maintained at a constant pressure. The sample flowed consecutively through the EPC, SP cell, MP cell, metering valve, and pump. A metering valve was used to regulate the outlet flow rate, which was dominated by an overrated pump.

### 2.2. Electronic Circuit

**Figure 2:**
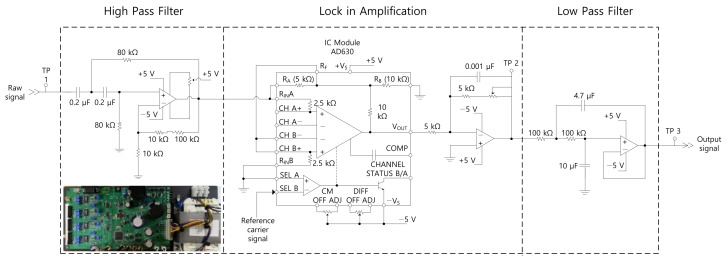
Electronic circuit diagram of a single signal processing channel. The circuit contains an HPF, a demodulator, an LPF, and op-amps at each stage. The HPF design included an 80 kΩ resistor and a 0.2 μF capacitor used to set a cut-off frequency of 10 Hz. The demodulated signal was obtained after HPF was connected to the input line of IC chip (AD630) with a gain of 2. The LPF comprised a 100 kΩ resistor and 4.7 μF and 10 μF capacitors used to set a cut-off frequency of 0.23 Hz. Only the DC component was extracted after removing random noise. The op-amps were configured at each stage for signal amplification. The typical amplification factor was 88.

**Figure 3:**
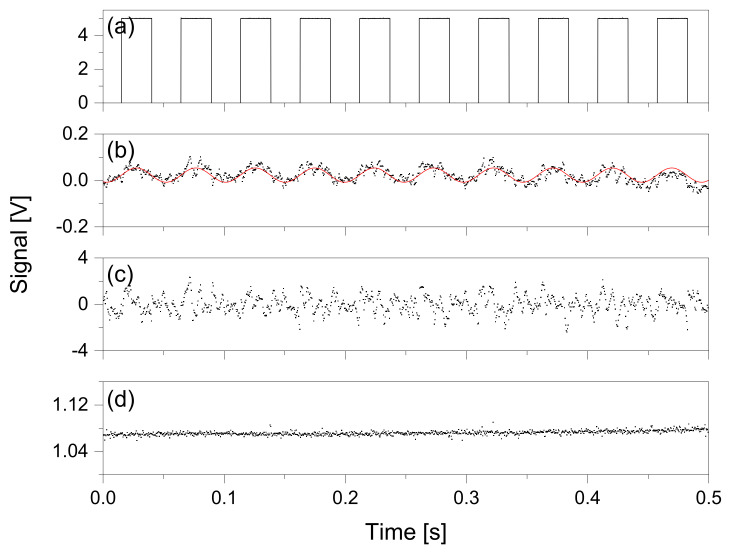
Signal processing in the measurement using the MP cell. (**a**) External reference TTL signal to generate the chopper at a repetition frequency of 20 Hz. (**b**–**d**) Time traces measured at PCB test points (TP) with an oscilloscope, measured at TP1, TP2, and TP3, respectively. (**b**) Modulated raw signal detected by the detector, with the non-linear fitted curve with sinusoidal function (red). The signal amplitude was 0.06 V (peak to peak), and the noise was calculated by the root mean square (rms) of the fit residual, while the signal to noise ratio (S/N) was 3.14. (**c**) Demodulated signal, where the waveform was identified by fitting with squared sinusoidal function. (**d**) Final DC signal which passes through the LPF. The signal size was 1.071 V, indicating a 17-fold increase when compared to the raw signal. The S/N was 277, indicating an 88-fold improvement when compared to the raw signal. The DC signal presented an accuracy of 0.72% (*k* = 2) for 0.5 s.

An optical chopper was used to modulate the collimated light source of the SiC which was powered by the constant-voltage power supply (24 V and 1.5 A). The chopper controller (Thorlabs, MC2000B-EC) generated a TTL signal with a repetition rate of 20 Hz, which was applied to the reference channel of the lock-in amplifier (LIA). D1, D2, and D3 all used the same type of pyroelectric sensor, which had a detectivity of 6 × 10^8^ $\mathrm{cm}\sqrt{\mathrm{Hz}}/W$ (at 10 Hz) and a modulation frequency range of 1–100 Hz. The raw signal was amplified using a preamplifier integrated with a pyroelectric sensor. A homemade electronic circuit was used to process the signals (Figure 2). The raw signal of each detector was fed to three identical but independent channels comprising a high-pass filter (HPF), LIA, and low-pass filter (LPF). An operational amplifier (op-amp) was used at each signal-processing stage to amplify the signals. The HPF was designed to have a cut-off frequency of 10 Hz by using a 0.2 μF capacitor and 80 kΩ resistor. The sinusoidal modulation floor of the raw signal (Figure 3b) was filtered using an HPF. Subsequently, a commercial integrated chip demodulator (Analog Devices, AD630) was used to form the LIA. The input signal was connected to the non-inverting input of a 2.5 kΩ resistor to minimize any errors produced by the input bias current. The input signal was connected to the ground through resistors, R_a_ (5 kΩ) and R_b_ 10 (kΩ), to form a closed-loop pre-amplifier with a gain of 2. The reference carrier signal was connected to the comparator to synchronize the phase-lock loop of the LIA unit with the optical chopper. The filtered and amplified signal (Figure 3c) was fed to the LPF, which comprises a 100 kΩ resistor and 4.7 μF and 10 μF capacitors. The high-frequency noise was filtered, and the final DC signal was removed (Figure 3d). The amplification factor of the homemade LIA unit was 88. The DC signal was then digitized by using an analog-to-digital converter (ADC, Texas Instruments, ADS1115) with a sampling rate of 500 samples/s and a vertical resolution of 16 bits. The digital signal was temporally stored on board the memory of a microcontroller unit (MCU, Atmel, ATmega128) and then transmitted to a PC using a UART communication protocol. The DC signals (0 V and 5 V) used to control the two FFMs were generated by the MCU and ADC. The wavelength of the tunable filter, i.e., the FPI, was tuned by using an application-specific integrated circuit (ASIC). The wavelength data were obtained using EEPROM, where a factory-generated look-up table for the post-calibration of wavelength was stored. The wavelength value transmitted to the PC using the UART communication protocol was estimated based on the FPI-derived voltage and measured temperature. DC power of 90 V was supplied to the FPI control board (Infratec, DP-C) and was regulated from 30 V to 90 V to tune the center wavelength, i.e., performed the length adjustment of the PZT integrated with the FPI. The voltage regulation was performed by transmitting commands via the MCU.

## 3. Experimental Section

### 3.1. Signal Processing

The signal traces were observed using an oscilloscope (Teledyne LeCroy, HDO6034, bandwidth = 350 MHz) at the test points on the PCB (TP1, TP2, and TP3 in Figure 2). Figure 3 depicts the time traces of the detection signals at each test point, which were recorded at 10,000 data samplings for 0.5 s. The chopper was operated at a frequency of 20 Hz with a reference TTL signal (Figure 3a). The raw signal was modulated using the same frequency as that of the chopper at 20 Hz, as shown in Figure 3b. This signal trace was fitted using nonlinear regression with a sinusoidal model function. The modulation depth red from the fitted curve (represented by red trace in Figure 3b) was 0.06 V peak to peak. The noise level was estimated using the root mean square (rms) of the residuals, where the SNR was 3.14. The noise in the low-frequency region of less than ~10 Hz was removed from the raw signal by passing it through the HPF. The chip modulator, i.e., the phase-sensitive detector, multiplies the square carrier signal and the sinusoidal raw signal; therefore, the time trace of the output of the chip demodulator is given as follows [23]:

(1) $$u_{PSD}\left(t\right)=\frac{2V_{s}V_{ref}}{\pi }\sum_{n=1}^{\infty }\frac{{\left(-1\right)}^{n+1}}{2n-1}\cos\left[\left(2n-2\right)\omega_{0}t-\theta \right]+\frac{2V_{s}V_{ref}}{\pi }\sum_{n=1}^{\infty }\frac{{\left(-1\right)}^{n+1}}{2n-1}\cos\left[2n\omega_{0}t+\theta \right]$$

where *V_s_* denotes the response of the PD, *V_ref_* denotes the amplitude of the reference signal, *ω*_0_ (= 2π*f*_0_) denotes the angular frequency of the detected signal, *f*_0_ denotes the synchronized frequency of the signal and reference signals, and *θ* denotes the phase shift between the raw and reference signals. Therefore, after the LPF (in this study, the cut-off frequency is ~0.23 Hz), the LIA output is given as follows:

(2) $$u_{LIA}=\frac{2V_{s}V_{ref}}{\pi }\cos\theta$$

The above equation yields a constant value assuming that the phase shift between the reference and raw signals remains constant. A 0° phase shift sets the maximum amplitude of the LIA output. As expected, the LIA and LPF units worked successfully to leave the DC component (Figure 3d). The noise pattern of the demodulated signal (Figure 3c) appeared to be similar to the fit residual of the raw signal (Figure 3b), which was amplified using the HPF-LIA units but discriminated in the LPF. The intensity of the processed signal at each stage was amplified using an op-amp. Under ideal phase-shift conditions, the amplitude of the resulting DC signal was obtained as 1.07 V, representing a 17-fold increase when compared to the raw signal, while a 277-fold increase was observed in the SNR of the DC signal, representing an 88-fold improvement when compared to the raw signal. The final DC signal presented an accuracy of 0.72% (*k* = 2). The slow drift of the DC component is caused by the drift of the light source.

### 3.2. Spectroscopy

**Figure 4:**
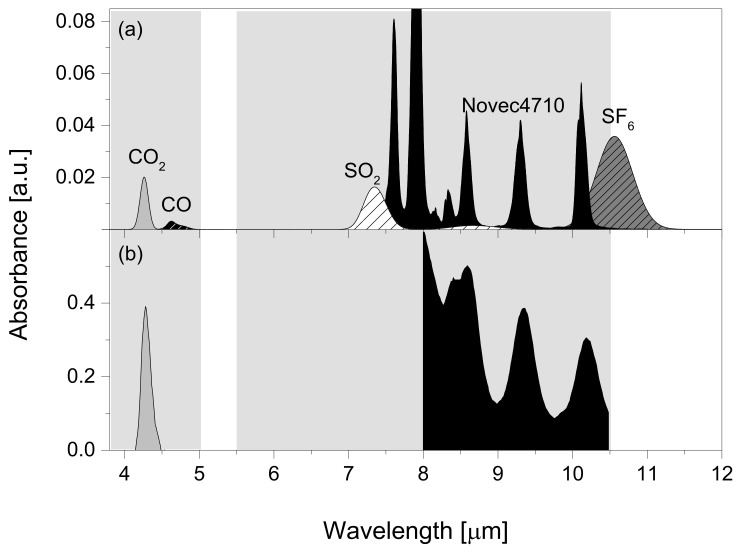
Wavelength tuning range of the detector and simulated absorption spectra. (**a**) Typical spectra of Novec-4710, CO, CO_2_, SO_2_, and SF_6_ detectable within the wavelength scan range of the spectrometer. The simulated spectra of CO, CO_2_, SO_2_, and SF_6_ were based on the HITRAN database. The simulation conditions were as follows: concentration of 50 ppmv, OPL of 10.5 m, cell injection pressure of 50 Torr, and resolution of 50 cm^−1^. The Novec-4710 spectrum was measured using an FTIR spectrometer, and the measurement conditions were as follows: resolution of 0.5 cm^−1^ and the same concentration, OPL, and cell injection pressure as the simulation conditions. (**b**) CO_2_ and Novec-4710 spectra measured while scanning the wavelength of the tunable filter. CO_2_ with concentration of 58.2% and Novec-4710 with concentration of 5.0% were injected into the SP cell with an OPL of 5 cm under an injection pressure of 100 Torr. Wavelength scanning was performed from 3.8 μm to 10.5 μm for the CO_2_ spectrum and from 8.0 μm to 10.5 μm for the Novec-4710 spectrum.

The TF-NDIR analyzer could scan wavelengths in the range of 3.8–10.5 μm, excluding 5.0~5.5 μm. Therefore, most gases can be measured with sufficient sensitivity, since the line strengths of most gases in this wavelength range exist and are always better than those in the NIR. For example, the absorption spectra of CO_2_, CO, SO_2_, Novec-4710, and SF_6_ were simulated using forward modeling based on the HITRAN database (Figure 4a). [24] The spectrum simulation was conducted for 50 ppmv in an N_2_ broadener with OPL of 10.5 m, total pressure of 50 Torr, and instrumental resolution (Gaussian) of 50 cm^−1^. However, owing to the unavailability of the spectral database of Novec-4710, an FTIR (Bruker, 125HR, spectral resolution = 0.5 cm^−1^) spectrometer was used for direct measurement, where the concentration, OPL, and cell pressure were identical to those of the simulation conditions. The only difference was that CO_2_ is a broadener. Nevertheless, the broadening effect may be insignificant because a single rotational line cannot be resolved due to the heavy molecular weight of Novec-4710, and instrumental line broadening dominates alien gas broadening for the band shape to not be altered under the given measurement conditions. Figure 4b depicts the absorption spectra of CO_2_ and Novec-4710, which are measured using a TF-NDIR analyzer. CO_2_ was measured by 58.2% CO_2_ in N_2_ in the SP cell (5 cm in length) at 100 Torr, whereas Novec-4710 was measured by 5.0% Novec-4710 in CO_2_ into the SP cell at 100 Torr. The measured spectra presented the same pattern as the simulated spectra. This example demonstrates that the developed TF-NRIR analyzer can be used to evaluate the mixing ratio of the Novec-4710/CO_2_ mixture and to ensure that the dielectric strength of the GIS is adequate instead of using gas chromatography which typically requires multiple sample injections and a long retention time [25].

## 4. Result and Discussion

**Figure 5:**
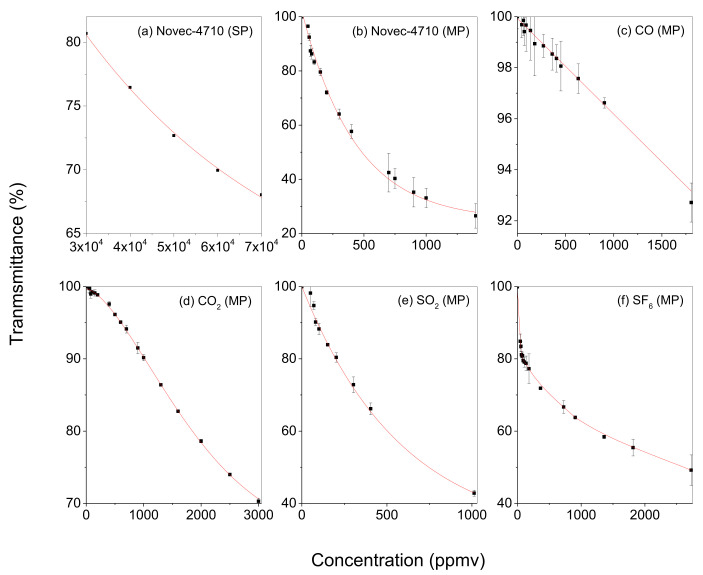
Transmittance according to concentration. The correlations between gas concentration and transmittance were verified at an OPL of (**a**) 5 cm and (**b**–**f**) 10.5 m. (**a**) The transmittance measured after injecting 3~7% of Novec-4710 into the SP cell with an OPL of 5 cm at 100 Torr. The transmittance measurement data were fitted by the exponential decay function based on the concentration and matched with R^2^ = 0.999. (**b**–**f**) The transmittance measured after injecting several dozen to several thousand ppmv of mixed gas with N_2_ as the background gas into the MP cell with an OPL of 10.5 m at 100 Torr. The measured components are (**b**) Novec-4710, (**c**) CO, (**d**) CO_2_, (**e**) SO_2_, and (**f**) SF_6_. For fitting, an exponential decay function was used for Novec-4710 and SO_2_, a linear function was used for CO, and a sigmoidal function was used for CO_2_. For SF_6_, the measurement points were connected by splining.

Quantitative analysis using spectroscopy is based on the Beer–Lambert law, which demonstrates a change in the radiant power when the radiant power in a beam of electromagnetic radiation passes through a cell containing a homogeneous mixture. The Beer–Lambert law can be expressed as follows:

(3) $$T=\frac{P\left(\lambda \right)}{P_{0}\left(\lambda \right)}=10^{-\varepsilon \left(\lambda \right)\cdot l\cdot c}$$

where P [W] denotes the transmitted light radiant power of the cell, $P_{0}\;\left[W\right]$ denotes the initial light radiant power, T denotes the transmittance, $\varepsilon \left(\lambda \right)\;\left[{\mathrm{cm}}^{2}/\mathrm{mol}\right]$ denotes the molar absorption coefficient at wavelength, λ, l [cm] denotes the OPL, and $c\;\left[\mathrm{mol}/{\mathrm{cm}}^{3}\right]$ denotes the molar concentration [26,27,28]. The transmittance (T) is the ratio of the initial light radiant power ($P_{0}$) to the vacant cell and light radiant power (P) transmitted through a gas cell filled with absorbing gas. Transmittance is complementary to the molar absorption coefficient, optical path length, and molar concentration. A long OPL enhances the sensitivity to perform trace-level detection in the case of trace-level concentration. Figure 5a depicts the correlation between concentration and transmittance when the sample is present inside the SP. The samples measured were Novec-4710 in CO_2_ mixtures with concentrations of 3, 4, 5, 6, and 7%. This measurement capability is required for testing the insulation power, which is primarily affected by the mixing ratio of Novec-4710/CO_2_ [29]. The cell was filled with the gas mixtures at 100 Torr, and EPC was used to maintain a constant cell pressure. The center wavelength was 8.6 μm, which is one of the peaks of the Novec-4710. Variations in the transmittance could be predicted well based on the variations in the Novec-4710 concentration, as fitted by the exponential decay function (R^2^ = 0.999) (Figure 5a). Figure 5b–f depict the correlation between the transmittance and the concentration of Novec-4710, CO, CO_2_, SO_2_, and SF_6_ in the MP. The transmittance was measured using 1000, 903, 1000, 1013, and 907 ppmv in N_2_ for each premixed gas. In the case of Novec-4710, the Novec-4710/CO_2_ mixture (at percent level) was diluted by N_2_ where the dilution ratio controlled the ratio of the flow rates of the Novec-4710/CO_2_ premix and high purity N_2_. The flow rates were adjusted by using two well-calibrated mass flow controllers (Brooks, 5850E). Then, the effective concentrations of the measurands were adjusted by controlling the total pressure of the premixed gases. The trace CO and SO_2_ measurements were conducted for demonstrating the detection of the side products of Novec-4710/CO_2_ and SF_6_, respectively, by arc or thermal decompositions at the switching electrode. The trace Novec-4710 and SF_6_ measurements demonstrated a leakage detection of the Novec-4710/CO_2_ and SF_6_. The leak rate of the Novec-4710/CO_2_ mixture, which fills the eco-friendly GIS, needs to be measured by the trace level of CO_2_ in air-free conditions, for instance, a well-sealed house isolated from the atmosphere. The data point depicted in Figure 5 were measured in a well-conditioned laboratory (with a temperature of 23–25 °C) and averaged based on three times repeated measurements; each measurement was averaged by 0.5 s. Data points, which have an error bar higher than ~1%, contained the uncertainty source attributed to the drift of the light source. For the fitting, an exponential decay function was used for Novec-4710 and SO_2_, a straight line for CO, and a sigmoidal function for CO_2_. For SF_6_, the measurement points were connected via the spline algorithm. Although this type of model function does not have a physical meaning, it suggests a calibration function for the quantitative analysis of the sampled gas. Table 2 lists the limits of detection (LOD) for each gas. The LOD was derived from a straight line fit using transmittance measured at concentrations below 100 ppmv. The LOD was calculated as follows [30,31]:

(4) $$\mathrm{LOD}=T\times S_{a}/b$$

where T denotes a student-T factor at a 99% confidence level, which is calculated with the degree of freedom, i.e., n − 1 (n denotes the used sample number), *S_a_* denotes the standard deviation of the fit residual, and *b* denotes the slope. Therefore, the LOD was estimated to be three times that of the noise over the sensitivity near the LOD level. The LOD of Novec-4710, CO, CO_2_, SO_2_, and SF_6_ was 54.7 ppmv, 112.8 ppmv, 118.1 ppmv, 69.5 ppmv, and 33.5 ppmv, respectively. The LOD of each gas was measured at the peak position presented in Figure 4. The LOD of Novec-4710 was measured at 8.6 μm because it is the most intense peak except for 8.0 μm, which was saturated at a concentration of 5.0%. The spectral interference between SF_6_/SO_2_ and Novec-4710 does not need to be considered for the diagnostics of the GISs, because SF_6_ and Novec-4710-based GISs are generally operated in separate sites. In the case of CO, which is one of the major side-products of the arc or thermal decomposition reactions of the Novec-4710/CO_2_ mixture in the eco-friendly GIS, it can be conjectured that the spectrum of CO is predominantly separated from that of the matrix CO_2_, based on the similarity in the measured and simulated spectra of CO_2_. In the simulated spectra, CO_2_ and CO were completely separated, indicating no interference in the NDIR measurement of trace CO in the Novec-4710/CO_2_ mixture. A slight variation in the center wavelength of the tunable filter, caused by temperature variation, can affect the drift in the detection sensitivity. For instance, the detection sensitivity is reduced out of the peak wavelength, leading to an increase in the LOD due to the increase in *b*.

Additionally, maintaining the drift of the light source is crucial for improving the measurement reproducibility. For the field measurement, the drift compensation might be required by adding a reference channel whose configuration is identical to that of the detection unit or the configurable optical filter unit with a pyroelectric sensor.

## 5. Conclusions

The NDIR-based gas detector comprises conventional optical filters and sensors. The FWHM of the optical BPF is several hundred nanometers, and can cover a single absorption band, implying that there are limitations in the detection of various components. In this study, three detectors attached to the FPI were used to develop an analyzer for the MIR region that can scan a width of approximately 6700 nm. The detection wavelength of the analyzer was 3.8~10.5 μm, excluding 5.0~5.5 μm, which enabled the detection of various gases within the spectral range. The analyzer had two OPLs (5 cm and 10.5 m) for a wide concentration range, from a leak level of dozens of ppmv to a Novec-4710/CO_2_ mixing ratio of several percent. The OAP mirrors were used to convert a point light source to a collimated beam, which was required to maintain a high beam quality in order to obtain an adequate throughput from the MP cell. The radiant power of the light source divided in a of ratio 75:25 (MP:SP) was measured at the same level. Furthermore, focusing was performed using an OAP mirror in the front part of the detector to enhance the detected radiant power, which resulted in a 10-fold increase. The HPF, LIA, and LPF were configured as a single channel and integrated into the PCB for signal processing. Three channels were operated independently for detection by type, and the detected signal was transmitted to the PC through the ADC/MCU. The precision of the measured signals was 0.72% (*k* = 2). Lastly, the demodulated DC signal exhibited a 17-fold increase in the signal size and an 88-fold improvement in the SNR when compared to the raw signal. The performance of the developed gas analyzer was evaluated by measuring the ppmv level of SF_6_ and the percent level of a Novec-4710/CO_2_ gas mixture, which is used as the dielectric gas of the GIS. SO_2_ and CO were also analyzed as byproducts of pyrolysis or high-voltage arcs. This analysis was significant because they are considered to be a key indicator for the health monitoring of the GIS. CO_2_ and CO were measured independently without interference, by comparing their measured and simulated spectra (Figure 4). This is significant because CO, which is a by-product of the Novec-4710/CO_2_ mixture in a high-energy arc, must be measured in the presence of the Novec-4710/CO_2_ mixture. In the SP cell, the transmittance and mixture ratio of Novec-4710/CO_2_ were highly correlated. Furthermore, high correlations were observed in the MP between the transmittance and ppmv concentration of Novec-4710, CO, CO_2_, SO_2_, and SF_6_. The LOD was 54.7 ppmv (Novec-4710), 112.8 ppmv (CO), 118.1 ppmv (CO_2_), 69.5 ppmv (SO_2_), and 33.5 ppmv (SF_6_), and is affected by the accuracy of the wavelength, white noise of the pyroelectric detector, and EPC accuracy. Particularly, the sensitivity and precision of the gas analyzer can be improved by improving the body temperature, wavelength accuracy, and light source drift. In this study, convection cooling was performed using a fan, and the performance of the analyzer was affected by variations in the laboratory environment. However, the thermoelectric cooling of the detectors and light source housing can enhance the measurement performance (precision and LOD). The multi-gas analyzer demonstrated in this study presents several advantages since it can be used to simultaneously monitor the operating status of two types of GISs based on SF_6_ and Novec-4710/CO_2_. The proposed TF-NDIR analyzer is a powerful diagnostic tool that can be implemented for various GISs in the introduction period of eco-friendly GIS to power grids. Additionally, the developed gas sensor is expected to be highly effective since air pollution monitoring stations have started to implement individual NDIR analyzers for each type of gas for various environmental pollutants. When compared to commercial devices, the TF-NDIR presented in this study exhibited a comparable LOD (tens of ppmv) and precision (0.75%, *k* = 2), but faced no limitations in selectivity. The addition of a reference channel to compensate for the drift of the light source is crucial in enabling the commercialization of field testing.

## Figures and Tables

**Table 1:** Selection of gas cell and detector based on the combination of two flip flop mirrors (FFM). FFM operates with two statuses: “on” for a beam passing through and “off” for a reflected beam. The FFM1 status primarily affects the gas cell selection, whereas the FFM2 status primarily affects the detector selection.

FFM 1	FFM 2	Passed Cell	Detector
on	on	SP	D3
on	off	SP	D1
off	on	MP	D3
off	off	MP	D1
on	anything	MP	D2

**Table 2:** Limit of detection (LOD) of each component in the optical configuration with an OPL of 10.5 m. The measurement wavelength of LOD was set to vary based on the molecular properties. The sensitivity of the detector varied based on the measurement wavelength, and the LOD was affected by the detector sensitivity.

Component	Wavelength [μm]	LOD [ppmv]
Novec-4710	8.6	54.7
CO	4.6	112.8
CO_2_	4.3	118.1
SO_2_	7.4	69.5
SF_6_	10.5	33.5

## Data Availability

Not applicable.
